# *AtMRP6*/*AtABCC6*, an ATP-Binding Cassette transporter gene expressed during early steps of seedling development and up-regulated by cadmium in *Arabidopsis thaliana*

**DOI:** 10.1186/1471-2229-8-22

**Published:** 2008-02-28

**Authors:** Stéphane Gaillard, Hélène Jacquet, Alain Vavasseur, Nathalie Leonhardt, Cyrille Forestier

**Affiliations:** 1 CEA, DSV, IBEB, Lab Echanges Membranaires & Signalisation, Saint-Paul-lez-Durance, F-13108, France; 2 CNRS, UMR 6191 Biol Veget & Microbiol Environ, Saint-Paul-lez-Durance, F-13108, France; 3 Aix-Marseille Université, Saint-Paul-lez-Durance, F-13108, France; 4 Institut de Biologie du Développement de Marseille-Luminy (IBDML), CNRS, UMR 6216; Case 907, Parc Scientifique de Luminy, 13288 Marseille Cedex 9, France

## Abstract

**Background:**

ABC proteins constitute one of the largest families of transporters found in all living organisms. In *Arabidopsis thaliana*, 120 genes encoding ABC transporters have been identified. Here, the characterization of one member of the MRP subclass, *AtMRP6*, is described.

**Results:**

This gene, located on chromosome 3, is bordered by *AtMRP3 *and *AtMRP7*. Using real-time quantitative PCR (RT-Q-PCR) and the GUS reporter gene, we found that this gene is essentially expressed during early seedling development, in the apical meristem and at initiation point of secondary roots, especially in xylem-opposite pericycle cells where lateral roots initiate. The level of expression of *AtMRP6 *in response to various stresses was explored and a significant up-regulation after cadmium (Cd) treatment was detected. Among the three T-DNA insertion lines available from the Salk Institute library, two knock-out mutants, *Atmrp6.1 *and *Atmrp6.2 *were invalidated for the *AtMRP6 *gene. In the presence of Cd, development of leaves was more affected in the mutants than wild-type plants, whereas root elongation and ramification was comparable.

**Conclusion:**

The position of *AtMRP6 *on chromosome 3, flanked by two other *MRP *genes, (all of which being induced by Cd) suggests that *AtMRP6 *is part of a cluster involved in metal tolerance, although additional functions in planta cannot be discarded.

## Background

Contamination of soil by agronomical and industrial activities, notably heavy metals, is a major problem for human health. In the past years, decontamination by plants (phyto-remediation) has been the subject of intensive research. Some heavy metals such as copper, iron and zinc are oligo-elements essential for plant development, however they can become toxic at higher concentrations. Conversely, non-nutrient metals such as cadmium (Cd), lead and mercury are potentially toxic even at very low doses. Nonetheless, their toxicity varies between plant species. For example, metal-tolerant plants are able to grow in highly contaminated soils. Mechanisms responsible for the uptake and storage of heavy metals in plants began to be understood [1]. First after mobilization of metal ions from soils, uptake of heavy metals occurs into root cells through more or less specific channels and/or transporters [2-4]. In a second phase occuring in the cytoplasm metal ions are associated with amino acids, organic acids, glutathione or longer glutathione-derived peptide, phytochelatins (PCs). When plants are exposed to Cd, an increase in PCs synthesis occurs and these PCs participate in the root to shoot translocation of Cd [5]. In a third phase, glutathione and PCs-Cd complexes are excluded from the cytosol into vacuolar or extra-cellular compartments by various transporters, among which are ABC transporters [6,7].

The ATP-binding cassette (ABC) superfamily is the largest family of transporters in living organisms, ranging from bacteria to humans [8-10]. In humans, ABC transporters have received considerable attention as their deficiency or mutations are associated with severe diseases such as cystic fibrosis and diabetes [11,12]. These transporters are able to carry various substrates, including ions, carbohydrates, lipids, xenobiotics, drugs and heavy metals [11,13-18]. In the *Arabidopsis *genome, 120 genes encoding ABC proteins have been identified [10], but for most of them, their function and substrates are still unknown. A number of ABC transporters were recently characterized for auxin and chlorophyll catabolites transport [19-23], pathogen and antibiotic resistance [24-27], detoxification of heavy metals [6,7,28,29], as well as for controlling water stress *via *anions and calcium channel regulation [30,31].

Fifteen members of the Arabidopsis ABC transporters belong to the multidrug resistance-associated protein (MRP) subfamily [32]. MRP proteins display two hydrophobic domains (TMD) containing six membrane spans and two hydrophilic, cytosolic, nucleotide binding domains (NBD) which are organized in pairs. In most of MRP proteins, an additional hydrophobic domain (TMD_0_, including 3 to 5 transmembrane spans) is present at the N-terminal part of the transporter. In most ABC transporters, the binding and subsequent hydrolysis of ATP at their NBD provides the energy required for substrate translocation across the membrane. Structurally, each NBD exhibits one 'Walker A' and one 'Walker B' motif which is endowed by all ABC members, as well as by other ATP-binding proteins, and a highly conserved C motif or ABC transporter signature, being located between both Walker sequences, which is specific to ABC transporters. Until now, five members of this subclass (AtMRP1 to AtMRP5) have been characterized and AtMRP1, AtMRP2 and AtMRP3 have been found to exhibit glutathione S-conjugate transport activity [19,33]. In the case of AtMRP2 and AtMRP3, an additive chlorophyll catabolites transport activity was reported [19,20]. Interestingly, AtMRP3 is also able to complement the loss of YCF1, which is an ABC transporter involved in Cd detoxification in yeast [20]. *In planta, AtMRP3 *is up-regulated by a Cd treatment [28,34], but the evidence that AtMRP3 is a Cd-transporter has not yet been obtained and to our knowledge there is no description of any *Atmrp3 *mutant in the literature till now. In addition, AtMRP4 and AtMRP5 are involved in the control of stomatal movements. More precisely AtMRP5 participates in the control of water loss *via *the regulation of anion and calcium channels [30,31,35-37]. Here, we report the expression pattern of *AtMRP6 *which is part of a cluster of three *MRP *genes co-regulated by Cd. Two T-DNA insertion mutants were isolated, and an increased sensitivity to Cd during early stages of development was observed in these two lines.

## Results

### cDNA isolation and protein organization

**Figure 1:**
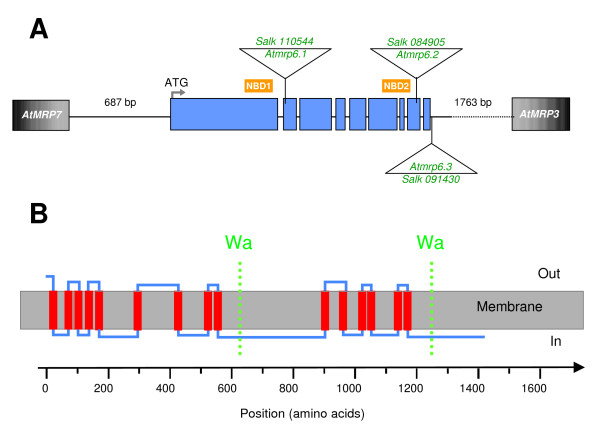
**Gene structure and protein topology**. **(A) **Genomic organization of the AtMRP6 gene (At3g13090) deduced from the cDNA. The 9 exons are represented by blue boxes. Triangles indicate the localization of T-DNA insertions in the three different insertion lines investigated. Position of the two nucleotide-binding domains is symbolized by the NBD boxes. The right and left flanking regions (*AtMRP3*, At3g13100 and *AtMRP7*, At3g13080) are represented by their intergenic distance. **(B) **Transmembrane domains were determined using the criteria proposed for classical membrane proteins [46]. It could be possible for the protein to exhibit an internal symmetry consistent with an even number of transmembrane helices, six in each half and a TMD_0 _of at least three transmembrane spans at the end terminal part. The X-Axis represents the amino-acids position along the protein sequence. Walker A domains are represented in both halves by the dotted lines.

*AtMRP6 *(according to the nomenclature proposed by Martinoia and col. [32]) was directly cloned by RT-PCR using MR06-NotStart and MR06R-StopNot oligonucleotide primers (table 1) and a full-length cDNA of 4398 bp was obtained (GenBank AY052368). Alignment of this cDNA with the genomic sequence (5200 bp) from chromosome III allowed us to deduce the genomic organization of the gene. *AtMRP6 *extends on a 5.2 kb fragment and is spaced out into 9 exons. (figure 1A). This cDNA was unstable in *Escherichia coli*, requiring a growth of the bacteria at 30°C in order to avoid mutations. Two other members of the MRP sub-family, *AtMRP7 *and *AtMRP3*, flank the *AtMRP6 *gene at its 5'- and 3'-end, respectively. All are oriented in the same transcription direction. *AtMRP7 *and *AtMRP3 *are the closest related genes to *AtMRP6 *and this cluster probably results from two successive gene duplications [38]. Mean percentage amino acid identities of AtMRP6 compared to AtMRP7 and AtMRP3 were 79.5% and 64.0%, respectively. The *AtMRP6 *cDNA contains an open reading frame, which encodes a 1466-aminoacids polypeptide with a predicted molecular weight of 164.4 kDa. Based on a Kyte and Doolittle hydropathy plot using ProtScale and depending on the software used for transmembrane spans prediction, AtMRP6 exhibits 11 (PredTmr algorithm) to 16 (PHDhtm algorithm) transmembrane helixes. When using Aramemnon, 16 different algorithms are compared and a consensus sequence is proposed with 12 transmembrane spans. However, in this prediction, downstream from the first nucleotide-binding domain, the second half of the protein exhibits only 4 transmembrane helixes whereas 6 transmembrane spans are usually found. HMMTop_V2 (very well-known and suitable for the analysis of ABC transporters) as well as Phobius, proposed a model with 15 transmembrane helixes. Taking into account the fact i) ABC transporters should have an internal symmetry; ii) the two NBD should be accessible to the cytosol; iii) the two NBD should not overlap the transmembrane region, we consider that the most probable model is the one presented in figure 1B, with at least 15 transmembrane helixes, two-halves of 6 transmembrane helixes and a TMD_0 _of at least 3 transmembrane spans.

**Table 1:** Name and sequence of the different primers used in this study

Primer name	Sequence [5'-3']
*MR06NotStart*	*AAATATGCGGCCGCTATAAAGTGAACATTTTGGTCAACACTCAGTTCCTGATGGA*
*MR06R-StopNot*	GACCAAGGTTGTGAATCTGATTATACACTTCTATTTACGCTTTT *ATAACTAGAAGAAATATGCGGCCGCTATAAA*
*AtMRP6-GFP_A*	*GCCCATGGTGCTGCATGGACTGACATGC*
*AtMRP6-GFP_C*	*GCTCCTCGCCCTTGCTCACCATGCTTCTTTTGGATTTGGATTC*
*AtMRP6-GFP_B*	*GAATCCAAATCCAAAAGAAGCATGGTGAGCAAGGGCGAGGAGC*
*Rev_fin_GFP+Not I*	*ATAGTTTAGCGGCCGCTTTACTTGTACAGCTCGTCC*
*MR06F-2500-Sbf1*	*CCTGCAGGTCCTTATCGTCTTCATCC*
*MR06R-1-Xma*	*CCCGGGCAGGAACTGAGTGTTGACC*

### *AtMRP6 *can be expressed in mammalian cells but not in yeast

In order to investigate the ability of AtMRP6 to transport classical substrates of MRPs proteins, heterologous expression of the cDNA was realized in both yeast and mammalian cells (HEK-293 cells).

**Figure 2:**
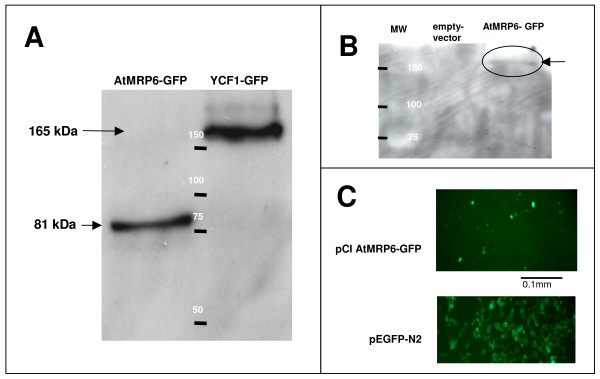
**Heterologous expression of AtMRP6 in yeast and mammalian cells**. **(A) **Immunodetection of GFP by western-blot analysis on total yeast proteins extracted by the trichloroacetic acid method. AtMRP6-GFP and YCF1-GFP lanes represent proteins extracted from yeast cells transformed by pYES2 *AtMRP6-GFP *and pYES2 *YCF1-GFP*, respectively. YCF1-GFP (165 kDa) was used as a positive control. Only the C-terminal part of AtMRP6 was preserved as a polypeptide of an apparent molecular mass of 81 kDa (theorical mass with the GFP: 192 kDa). **(B) **Immunodetection of GFP by western-blot analysis of HEK-293 cell proteins extracted by the RIPA buffer (50 mM Tris-HCl pH 7.4, 150 mM NaCl, 1 mM EDTA, 1% triton, antiproteases coktail). Empty-vector and AtMRP6-GFP lanes represent total proteins extracted from HEK-293 cells transfected by jetPEI with pCi and pCi AtMRP6-GFP, respectively. **(C) **Corresponding cells expressing AtMRP6-GFP in HEK-293 cells observed under fluorescence microscopy (excitation was performed at 488 nm, emission collected at 510 nm). As a control, cells expressing only GFP (pEGFP-N2) are presented in the lower panel.

EGFP was fused at the C-terminal part of AtMRP6 to localize its expression in both expression systems. Particular attention was dedicated to the integrity of plasmids due to the instability of *AtMRP6 *in *E. coli*. As shown in figure 2, a weak expression of the full size transporter was observed in HEK-293 cells. In yeast, the plasmid was intact but the protein underwent a maturation step, leading to a truncated version of the transporter (figure 2A). In these conditions, no complementation of the Δ*ycf1 *mutant by *AtMRP6-GFP *was observed (data not shown). In HEK-293 cells, AtMRP6-GFP was fully translated (figure 2B) but its expression level was low due to a weak yield of transfection and cellular expression (figure 2C), compared for instance to the GFP control (data not shown). Cell survival experiments conducted in the presence of exogenous Cd in the culture medium did not allow us to distinguish vector-transformed cells from cells expressing AtMRP6 (data not shown).

### *AtMRP6 *promoter-GUS fusion is essentially expressed in seedlings

**Figure 3:**
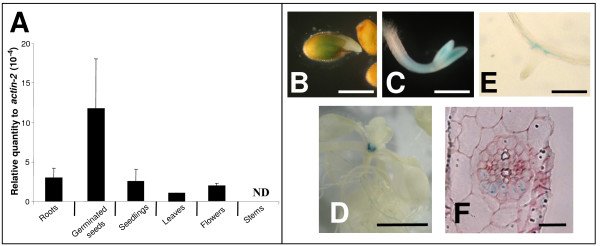
***AtMRP6 *gene expression determined by RT-Q-PCR and promoter GUS analysis**. **(A) **Quantification of *AtMRP6 *expression level by real-time quantitative PCR using mRNA extracted from various tissues or developmental stages. Values from three independent experiments are expressed relatively to actin-2 gene levels. **(B-F) **Activity of the *uidA *reporter gene in transgenic Arabidopsis plants expressing p*AtMRP6-GUS *fusion at different stages of development : germinated seeds after 24-hr **(B)**, seedling with closed cotyledons after 48-hr **(C)**, seedlings showing the apical meristem **(D)**, emergence of a secondary root **(E)**, root radial section **(F)**. (Scale bar corresponds to 0.5 mm in B and C, 0.5 cm in D, 0.2 mm in E and 50 μm in F).

*AtMRP6 *gene expression was determined by RT-Q-PCR in different tissues. As shown in figure 3A, *AtMRP6 *transcripts were principally detected in seedlings but at a very low level compared to the actin-2 gene. Expression was also found in roots, leaves and flowers but was absent from stems. This data was confirmed by analysis of independent homozygous transgenic lines expressing the β-glucuronidase reporter gene under the control of two different promoter regions of *AtMRP6*, one corresponding to the intergenic region (687 pb), the other corresponding to a 2.5 kb promoter region overlapping the ORF of *AtMRP7*. Transgenic plants expressing both constructions exhibited the same expression pattern. The GUS reporter gene was observed in germinating seeds (figure 3B), in young seedlings essentially in cotyledons (figure 3C), in more developed seedlings at the base of leaves and in the apical meristem (figure 3D). Expression was also detected in lateral root primordia (figure 3E), restricted to pericycle cells, which are found opposite the xylem pole on the side where lateral roots initiate (figure 3F).

### *AtMRP6 *is up-regulated by H_2_O_2 _and Cd exposure

**Figure 4:**
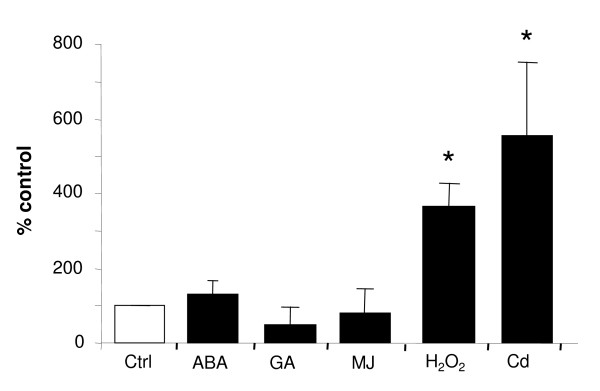
**Modulation of *AtMRP6 *gene expression level determined by quantitative real-time PCR in response to different stress conditions**. Variation of *AtMRP6 *gene expression in seedlings treated with different hormones (100 μM, 12-hr), after an oxidative stress (10 mM H_2_O_2_, 12-hr) or in roots of 3–4 week-old plants after Cd exposure (5 μM, 30-hr). (ABA: abscissic acid, GA: gibberillic acid, MJ: methyl jasmonate). Values from three independent experiments are expressed as percentage of control (untreated plants). (* : P < 0.05, t-test).

In order to determine in which process *AtMRP6 *could be involved, its expression level in response to numerous stresses was investigated by RT-Q-PCR in Arabidopsis plantlets. A significant variation of *AtMRP6 *expression level was observed after hydrogen peroxide treatment but not in response to hormones (brassinosteroid, abscisic acid and analogous-compounds, gibberillic acid or methyl jasmonate, figure 4) or to salt or cold stress (data not shown). Concomitantly by a transcriptomic analysis of genes regulated by Cd [39], we observed that *AtMRP6 *was one of the most induced ABC transporter genes. Such an up-regulation by Cd was confirmed by RT-Q-PCR, *AtMRP6 *being up-regulated in roots after a 30-hr exposition to 5 μM Cd (figure 4).

### Isolation and characterization of *Atmrp6 *knockout plants

**Figure 5:**
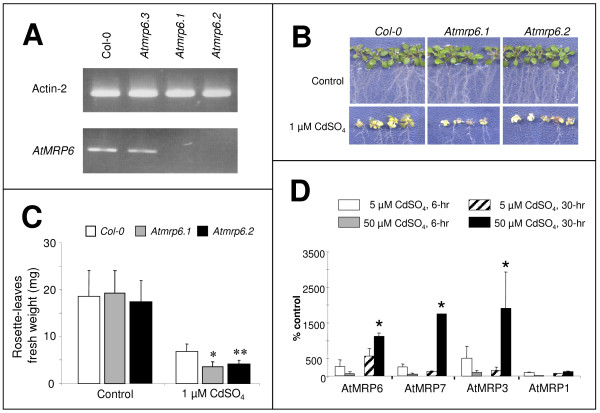
**Isolation, phenotypic characterization of *AtMRP6 *knock-out plants and co-regulation of the *AtMRP3, 6, 7 *genes cluster**. **(A) **Detection of *AtMRP6 *transcripts in the different T-DNA insertion lines determined by RT-PCR experiments on total RNAs isolated from roots of the different genotypes, using specific primers downstream from the insertions. (As a control, RT-PCR was performed with actin-2 primers.) (B) Growth of wild-type (Col-0), Atmrp6.1, and Atmrp6.2 mutant plants on agar plates, 21 days after germination, in the presence/absence of 1 μM CdSO4 (C) Cadmium sensitivity of Atmrp6.1 and Atmrp6.2 mutant plants measured as the rosette-leaves fresh weight. Bars correspond to the mean (± SEM) of eight agar-plate dishes from four independent experiments. In each agar-plate (with or without cadmium), 15 plants per genotype were analyzed. (D) Comparative expression of AtMRP1, 3, 6 and 7 genes in roots in response to cadmium. Plants were treated with CdSO4 in hydroponic conditions according to times and concentrations given in the caption. mRNAs were extracted and RT-Q-PCR were performed using specific primers for the three different genes of the cluster (AtMRP3, AtMRP6, AtMRP7) and with AtMRP1 (At1g30400) as a control. (C-D) Values from independent experiments are expressed as percentage of control (untreated plants). (** : P < 5e-3, * P < 8e-3, t-test).

In order to elucidate the function of *AtMRP6*, three T-DNA insertion knockout lines (figure 1A) were isolated from the SALK Institute collection: *Atmrp6.1 *(SALK #110544), *Atmrp6.2 *(SALK #084905) and *Atmrp6.3 *which are located downstream of the stop codon (SALK #091430). Since no full-length mRNA was detected in either *Atmrp6.1 *or *Atmrp6.2*, they were selected for further analysis. Amplification of the full messenger was obtained by RT-PCR in the case of the *Atmrp6.3 *mutant (figure 5A).

Growth and development of wild type plants as well as T-DNA KO plants (*Atmrp6.1, Atmrp6.2*) were similar when phenotypes were screened under various conditions such as sugar stress, oxydative stress (H_2_O_2_), hormones (brassinosteroid, 1-naphtaleneacetic acid, abscissic acid, salicylic acid), continuous light or darkness, or in the presence of calcium channels inhibitors known to interfere with Cd entry into the plant (data not shown, [4]). In hydroponic conditions, wild type *Columbia *ecotype (C*ol-0*), *Atmrp6.1 *and *Atmrp6.2 *KO mutant plants were exposed to 5 or 50 μM CdSO_4_, conditions that triggered an up-regulation of *AtMRP6 *(figure 4). For all plant genotypes, similar Cd contents were found by ICP-AES analysis in roots and leaves as well as similar GSH, γ-EC and phytochelatin contents determined by HPLC. Finally, all genotypes exhibited an equivalent resistance to Cd in terms of root growth and development (data not shown). Since the expression of *AtMRP6 *was essentially pronounced in seedlings (figure 3C–D), investigation of Cd effects was evaluated in *Atmrp6.1 *and *Atmrp6.2 *seedlings when seeds were directly sown on a Cd-contaminated medium. Three weeks after germination, root elongation and ramification in the absence or presence of 1–5 μM CdSO_4_were equivalent in all plant genotypes. However, *Atmrp6.1 *seedlings were more affected than control plants, notably at shoot level (figure 5B). In the absence of Cd, the fresh weight of *Atmrp6.1*, *Atmrp6.2 *and wild type rosette-leaves from seedlings were similar (20.4 ± 5.1 mg, 19.5 ± 2.9 mg and 19.6 ± 5.0 mg, respectively). Conversely, after Cd treatment, the fresh weight of *Atmrp6.1 *and *Atmrp6.2 *seedlings were significantly lower compared to wild-type (3.7 ± 1.2, 4.3 ± 0.8, and 6.9 ± 1.6, respectively) (figure 5C; mean of 4 independent experiments, 2 replicates per experiment). This reduction in fresh weight of the mutants was not accompanied by a change in Cd, GSH, γ-EC or phytochelatin content.

Thus, it can be concluded that invalidation of *AtMRP6 *increases Cd-sensitivity of seedlings. The possibility of an eventual functional redundancy within the *AtMRP3/AtMRP6/AtMRP7 *cluster was investigated. Since it had already been demonstrated that *AtMRP3 *is induced by Cd [28], we examined comparatively in wild type plants the expression levels of the three MRP genes belonging to the cluster, together with *AtMRP1 *as a control. As shown in figure 5D, the expression of the three genes was up-regulated by Cd in plant roots, whereas the expression level of *AtMRP1 *remained unchanged. The likely gene duplication at the basis of the *AtMRP3/AtMRP6/AtMRP7 *cluster [38] led us to investigate the expression level of *AtMRP3 *and *AtMRP7 *in the *Atmrp6*.1 mutant genetic background at the seedling stage of development. Whatever the presence or absence of Cd, no significant difference in *AtMRP3 *and *AtMRP7 *expression levels was observed. Therefore, invalidation of *AtMRP6 *was not correlated with an over-expression of *AtMRP3 *or *AtMRP7*.

## Discussion

ABC transporters, especially from the MRP subfamily, are frequently involved in the detoxification of various xenobiotics, among which, heavy metals are found. Here, we tried to decipher the function of a previously uncharacterized *A. thaliana *gene, *AtMRP6*, which is flanked by two other *MRPs *gene on chromosome III, *AtMRP3 *and *AtMRP7*.

Analysis of *AtMRP6 *gene expression by RT-Q-PCR as well as by promoter GUS analysis, demonstrated that this gene is weakly expressed and has a restricted pattern of expression, mainly in germinating seeds and seedlings. Subcellular localization of AtMRP6 *in planta *was attempted through two different approaches. First, CaMV35s transgenic plants expressing *AtMRP6-GFP *were generated. Strikingly, whereas empty vector and *AtMRP6 *antisens plants were easily obtained, it was never the case for the sense construction, probably indicating a toxicity of this gene product under over-expressing conditions. As an alternative way to address the localization of the transporter, mesophyll cell protoplasts were transfected with *AtMRP6-GFP *by the classical polyethylene glycol method. No fluorescence could be observed in these conditions whereas, in control cells expressing the GFP alone, fluorescence was detected in the cytoplasm and in the nucleus. The subcellular localization of AtMRP6 could not be determined however, our experiments highlighted the difficulties when working with this gene. In addition, heterologous expression of transporters in yeast constitutes an elegant approach to screening for complementation of various mutants and also to perform flux experiments with radiolabelled compounds. In the case of *AtMRP6*, no complementation of the Δ*ycf1 *mutant could be obtained in this study: AtMRP6 being truncated (figure 2A). We assume that this truncation of the protein was probably due to a toxicity of the transporter for the host. The development of such host toxicity is also consistent with an almost systematic mutation of the corresponding plasmid that occurred in bacteria at 37°C. When looking for an alternative expression system for AtMRP6, HEK-293 cells were transfected. As shown in figure 2B–C, AtMRP6 expression was successfully obtained. However, despite many efforts (assays with various plasmids such as pCi, pcDNA6 or pEGFP, optimization of the Kozak sequence, use of different cationic lipid transfection reagents), the yield of expression was too weak to initiate any flux experiment.

Results obtained in this study by RT-Q-PCR (figure 5D) and within a previous transcriptomic analysis [39], demonstrate that *AtMRP6 *expression is up-regulated in roots within 30-hr by 5 μM Cd. Interestingly, not only *AtMRP6*, but the three members of the gene cluster were also up-regulated by after Cd exposition. These results are in accordance with an enhanced level of both *AtMRP3 *and *AtMRP6 *transcripts, reported previously in cDNA microarray experiments [34]. It has already been reported that *AtMRP3 *can be important in Cd detoxification since its heterologous expression in the yeast strain deprived of *ycf1 *restores Cd tolerance [20]. However, in Arabidopsis, despite the fact that Cd-related induction of *AtMRP3 *is correlated with Cd uptake after a short metal exposure [28], whether AtMRP3 is involved in Cd transport or in the detoxification of toxic compounds produced after the metal stress awaits future studies. In the case of *AtMRP7*, very little data is available about its tissue expression [38] and its function is still unknown. A fourth gene, located upstream of the MRP cluster, is also up-regulated in roots by Cd treatment: it encodes a mitochondrial-localized serine acetyl-transferase, SAT3 or serat2.2 (At3g13110; [40]). This enzyme catalyzes the formation of O-acetyl-Ser from L-Ser and acetyl-CoA, which is used in cysteine synthesis, an important component of glutathione. Expression of the bacterial enzyme in tobacco led to an increase in cysteine and glutathione contents [41]. Moreover, the high activity of SAT is associated with nickel tolerance in *Thlaspi *nickel hyper-accumulators [42] suggesting a major role of SAT in heavy metal resistance. Recently, expression of SAT3 has been achieved in tobacco; however no experiments have been performed in relation to Cd [43]. All these results suggest that these four genes (*AtMRP3, AtMRP6, AtMRP7 *and *SAT3*), oriented in the same transcription direction on chromosome III, are members of a Cd-responding cluster. This hypothesis is also supported by the fact that all these genes are up-regulated by a Cd treatment into the same organ (roots) and in the same time scale (24-hr for SAT3, [40]; 30-hr for the three MRP genes). Identification of such Cd-responsive elements would be useful in the context of phytoremediation strategies either to drive the expression of cadmium-transporter or reporter genes that might be used as biosensors of contaminated soils.

At the sight of the expression pattern of this gene (figure 3), a phenotype was expected at root level in T-DNA KO lines. One cannot exclude that the neighboring MRP genes might complement the deletion of *AtMRP6*. For this reason, the expression levels of *AtMRP3 *and *AtMRP7 *were compared in wild type plants and in *Atmrp6 *genetic backgrounds. No significant difference in their expression levels was detected in the presence or in the absence of cadmium (data not shown). Thus, it is possible that if a mechanism of gene compensation is taking place in *Atmrp6 *KO plants, it involves (an)other gene(s) than *AtMRP3 *and *AtMRP7 *or that the basal levels of expression of *AtMRP3/7 *are sufficient to compensate for the absence of *AtMRP6*. Alternatively, these two genes could be up-regulated in the few cells expressing *AtMRP6 *in roots without significantly affecting their global root-expression level. The screening of several dozen conditions to observe a phenotype for *Atmrp6 *KO plants allowed us to show that, in the presence of Cd, the deletion of *AtMRP6 *has a small but significant impact on the development of primary leaves whereas roots elongation and ramification were unaffected. This phenotype was lost in 3- to 5-week-old plant, probably because at this developmental stage, Cd translocation from root to shoot is much lower, as already reported for AtMRP3 [34].

## Conclusion

We have shown that *AtMRP6*, *AtMRP3 *and *AtMRP7*, as well as *SAT3*, are part of a Cd-regulated gene cluster. The narrow expression profile of the *AtMRP6 *gene in the plant, essentially during the first step of seedling development might explain the discrete phenotype observed in T-DNA KO lines and is more consistent with a function of this transporter in plant growth/development rather than in Cd detoxification. If our results demonstrate that *AtMRP6 *is part of a cluster involved in metal tolerance, and that invalidation of this gene leads to a higher susceptibility of young seedlings, the precise function of this transporter in the plant will remain to be determined.

## Methods

### Plant materials, growth conditions and treatments

*Arabidopsis thaliana *T-DNA insertion knockout mutants of *AtMRP6 *(At3g13090) from the Salk Institute Library (Salk #110544, Salk #091430 and Salk #084905) were obtained from the NASC European Arabidopsis Stock Center (Nottingham, GB).

Surface-sterilized seeds (using 70% ethanol containing 0.04% SDS) were plated on agar solidified nutrient solution containing 805 μM Ca(NO_3_)_2_, 2 mM KNO_3_, 60 μM K_2_HPO_4_, 695 μM KH_2_PO_4_, 1.1 mM MgSO_4_, 20 μM FeSO_4_, 20 μM Na_2_EDTA, 74 nM (NH_4_)Mo_7_O_24_, 3.6 μM MnSO_4_, 3 μM ZnSO_4_, 9.25 μM H_3_BO_3_, 785 nM CuSO_4_, supplemented with 1% sucrose and 0.8% agar (SNS solution). After 2 to 3 days at 4°C, agar plates were cultivated under a 8-hr light period at 23°C (150 μmol m^-2 ^s^-1^) – 16-hr dark period at 19°C (70% relative humidity).

### cDNAisolation and subcloning in expression systems

Total RNAs from Arabidopsis plantlets were extracted by the Trizol™ method. Complementary DNAs were synthesized by using the First-Strand cDNA Synthesis Kit according to the manufactor's instructions (Amersham). PCR were realized using a high fidelity Taq polymerase with different primers MR06-NotStart and MR06R-StopNot showed in table 1. The NotI-flanked PCR product was cloned in the pCR-XL-Topo from Invitrogen^® ^and sequenced. The *AtMRP6 *cDNA sequence has been deposited in GenBank under the accession number AY052368. In order to localize AtMRP6, the C-terminal part of the cDNA was epitope-tagged with GFP. The plasmids pEGFP-N2 (from BD Biosciences^®^) and pCR-XL-AtMRP6 were used to generate the AtMRP6-EGFP-N2 fusion by the "splicing by overlap extension" technique as already described [44]. For this purpose, primers used were AtMRP6-GFP_A, AtMRP6-GFP_C, AtMRP6-GFP_B, and Rev_fin_GFP+NotI (table 1). The different sub-clonings from the pCR-XL-Topo AtMRP6-GFP to the yeast vector pYES2 (Invitrogen^®^) and the mammalian expression vector pCI (Promega^®^) were realized by a single restriction with NotI.

### Generation of *AtMRP6*::GUS lines

Two *AtMRP6 *promoters, corresponding to the intergenic region (687 bp) and to a 2511 bp sequence upstream of the start codon, were amplified on genomic DNA from Col-0 using specific primers (table 1) inserting SbfI and XmaI restriction sites and with PyroBest taq polymerase (Takara). PCR products were cloned in pGEM-T easy vector and verified by sequencing. SbfI-XmaI fragments were then inserted in pBI101 plant vector opened with the same enzymes. *Arabidopsis thaliana *Col-0 plants were transformed using *Agrobacterium tumefaciens*. Seedlings were selected on 30 μM kanamycin plates and six independent lines for each construction exhibiting a similar GUS pattern were selected.

### GUS staining

Plants or seedlings were pre-fixed in ice-cold 90% acetone for 20 min, washed with water and then with a 50 mM sodium phosphate buffer, pH 7.2. Tissues were incubated in the staining solution (50 mM sodium phosphate buffer, pH 7.2, 0.1% Triton ×-100, 0.5 mM potassium ferrocyanide, 0.5 mM potassium ferricyanide, containing 2 mM 5-bromo-4-chloro-3-indolyl-β-D-glucuronide (X-Gluc) overnight at 37°C. Stained samples were fixed in FAA (50% ethanol, 5% acetic acid, 3.7% formaldehyde) for one hour at room temperature, and progressively dehydrated. Cross-sections were obtained from dehydrated samples embedded in Technovit 7100 (Kulzer, Wertheim, Germany).

### Identification of *Atmrp6 *knockout mutants

Homozygous T-DNA insertion knockout mutants of *AtMRP6 *(At3g13090) were identified from SALK #110544 (*Atmrp6.1*), SALK #084905 (*Atmrp6.2*) and SALK #091430 (*Atmrp6.3*) seeds were obtained from the NASC (Nottingham, GB). A corresponding wild-type for each mutant was identified in the lineage of heterozygous T-DNA insertion mutants and were designated as Col-0 in the following. The T-DNA insertion site was confirmed by DNA sequencing. The presence of only one T-DNA insertion site was determined by Southern-blot as well as by segregation analysis of plantlets on 30 μM kanamycin.

### Real-Time quantitative RT-PCR

Total RNA was extracted from leaves, roots, stems, flowers, seedlings and germinating seeds, using Trizol^® ^according to the manufacturer's instruction (Invitrogen). Genomic DNA was removed from the samples using Dnase I (Ambion). Reverse transcription was performed using the First Strand cDNA Synthesis kit (Amersham) and an oligo-dT primer. PCRs were carried out using the SYBR Green Mix (Takara) in an optical 96-wells plate with the ABI PRISM 7900HT Sequence Detection System (Applied Biosystems). Specific primers for each gene were designed using the LightCycler Probe Design Software (Roche). The presence of a single amplicon in each PCR reaction was confirmed by dissociation curves and by loading on agarose gel. Standard curves were derived from reactions with *actin-2 *(At5g09810) specific primers, and a dilutions' series of cDNA templates. Relative quantity of transcripts analysed in each RNA sample was normalized to the standard curve and the mean value was calculated from three to four independent replicates.

### Cd treatment

For early Cd exposure, seeds were sown directly on agar plates containing 1 or 5 μM CdSO_4_. A longer vernalisation period of 4 days was used and seedlings were grown in a 14-hr light, 21°C, 10-hr dark, 18°C cycle for 21 days. Leaves were harvested and fresh weights were determined. Cd and thiol contents were measured by ICP-AES and by HPLC, respectively.

For late Cd treatment, 3–4 weeks old plants grown on sand were transfered in hydroponic conditions in a similar light/dark period at 23°C/19°C respectively, 250 μmol.m^-2^.s^-1 ^and 75% relative humidity. Cd treatments were carried out by adding 5 or 50 μM CdSO_4 _in nutrient solution for 6, 24 or 30 h as previously described [39]. Shoots and roots were harvested separately and supplied for Cd quantification by ICP-AES (6-hr and 30-hr) or for thiols measurement by HPLC (30-hr).

### Determination of Cd content

Fresh leaves, roots and seedlings from Cd-treated and untreated plants were dried 72-hr minimum at 50°C and mineralized in 70% HNO_3 _at 210°C for 10 min. The Cd concentration in the solution was determined using inductively coupled plasma optical emission spectroscopy (ICP-AES Vista MPX). Concentrations were normalized according to the dry weight of samples.

### GSH, γ-EC and Phytochelatin levels

GSH, γ-EC and PC levels in roots and leaves of Cd-treated and untreated *Atmrp6.1 *and *Atmrp6.2*, and corresponding wild-type plants were determined using 50 μg of plant material by HPLC analysis of monobromobimane-labeled compounds as previously described [45]. GSH, γ-EC and PC were quantified as nmol of thiol equivalents.

## Authors' contributions

SG carried out the molecular biology studies, the isolation and analyses of GUS-reporter lines. He carried out the isolation of mutants, characterized their phenotype and performed the statistical analysis. HJ carried out the yeast and mammalian cell studies and performed the cloning of the cDNA. AV contributed in the design of the study. NL carried out with SG the molecular analysis of transgenic plants and the transient transfection in protoplasts. CF was in charge of design and coordination of the study. SG, HJ and CF wrote the manuscript together. All authors read and approved the final manuscript.

## References

[B1] Clemens S, Palmgren MG, Kramer U (2002). A long way ahead: understanding and engineering plant metal accumulation. Trends Plant Sci.

[B2] Korshunova YO, Eide D, Clark WG, Guerinot ML, Pakrasi HB (1999). The IRT1 protein from Arabidopsis thaliana is a metal transporter with a broad substrate range. Plant Mol Biol.

[B3] Vert G, Briat JF, Curie C (2001). Arabidopsis IRT2 gene encodes a root-periphery iron transporter. Plant J.

[B4] Perfus-Barbeoch L, Leonhardt N, Vavasseur A, Forestier C (2002). Heavy metal toxicity: cadmium permeates through calcium channels and disturbs the plant water status. Plant J.

[B5] Gong JM, Lee DA, Schroeder JI (2003). Long-distance root-to-shoot transport of phytochelatins and cadmium in Arabidopsis. Proc Natl Acad Sci USA.

[B6] Kim DY, Bovet L, Kushnir S, Noh EW, Martinoia E, Lee Y (2006). AtATM3 is involved in heavy metal resistance in Arabidopsis. Plant Physiol.

[B7] Kim DY, Bovet L, Maeshima M, Martinoia E, Lee Y (2007). The ABC transporter AtPDR8 is a cadmium extrusion pump conferring heavy metal resistance. Plant J.

[B8] Higgins CF (1992). ABC transporters: from microorganisms to man. Annu Rev Cell Biol.

[B9] Van Veen HW, Konings WN (1997). Multidrug transporters from bacteria to man: similarities in structure and function. Semin Cancer Biol.

[B10] Garcia O, Bouige P, Forestier C, Dassa E (2004). Inventory and Comparative Analysis of Rice and Arabidopsis ATP-binding Cassette (ABC) Systems. J Mol Biol.

[B11] Gadsby DC, Vergani P, Csanady L (2006). The ABC protein turned chloride channel whose failure causes cystic fibrosis. Nature.

[B12] Gloyn AL, Siddiqui J, Ellard S (2006). Mutations in the genes encoding the pancreatic beta-cell K_ATP _channel subunits Kir6.2 (KCNJ11) and SUR1 (ABCC8) in diabetes mellitus and hyperinsulinism. Hum Mutat.

[B13] Ehrmann M, Ehrle R, Hofmann E, Boos W, Schlösser A (1998). The ABC maltose transporter. Mol Microbiol.

[B14] Borst P, Zelcer N, Van Helvoort A (2000). ABC transporters in lipid transport. Biochim Biophys Acta.

[B15] Pighin JA, Zheng H, Balakshin LJ, Goodman IP, Western TL, Jetter R, Kunst L, Samuels AL (2004). Plant cuticular lipid export requires an ABC transporter. Science.

[B16] Deeley RG, Westlake C, Cole SP (2006). Transmembrane Transport of Endo- and Xenobiotics by Mammalian ATP-Binding Cassette Multidrug Resistance Proteins. Physiol Rev.

[B17] Piddock LJ (2006). Multidrug-resistance efflux pumps – not just for resistance. Nat Rev Microbiol.

[B18] Sipos G, Kuchler K (2006). Fungal ATP-binding cassette (ABC) transporters in drug resistance & detoxification. Curr Drug Targets.

[B19] Lu YP, Li ZS, Drozdowicz YM, Hortensteiner S, Martinoia E, Rea PA (1998). AtMRP2, an Arabidopsis ATP binding cassette transporter able to transport glutathione S-conjugates and chlorophyll catabolites: Functional comparisons with AtMRP1. Plant Cell.

[B20] Tommasini R, Vogt E, Fromenteau M, Hortensteiner S, Matile P, Amrhein N, Martinoia E (1998). An ABC-transporter of Arabidopsis thaliana has both glutathione-conjugate and chlorophyll catabolite transport activity. Plant J.

[B21] Geisler M, Blakeslee JJ, Bouchard R, Lee OR, Vincenzetti V, Bandyopadhyay A, Titapiwatanakun B, Peer WA, Bailly A, Richards EL, Ejendal KF, Smith AP, Baroux C, Grossniklaus U, Muller A, Hrycyna CA, Dudler R, Murphy AS, Martinoia E (2005). Cellular efflux of auxin catalyzed by the Arabidopsis MDR/PGP transporter AtPGP1. Plant J.

[B22] Lin R, Wang H (2005). Two Homologous ATP-Binding Cassette Transporter Proteins, AtMDR1 and AtPGP1, Regulate Arabidopsis Photomorphogenesis and Root Development by Mediating Polar Auxin Transport. Plant Physiol.

[B23] Terasaka K, Blakeslee JJ, Titapiwatanakun B, Peer WA, Bandyopadhyay A, Makam SN, Lee OR, Richards EL, Murphy AS, Sato F, Yazaki K (2005). PGP4, an ATP Binding Cassette P-Glycoprotein, Catalyzes Auxin Transport in Arabidopsis thaliana Roots. Plant Cell.

[B24] Consonni C, Humphry ME, Hartmann HA, Livaja M, Durner J, Westphal L, Vogel J, Lipka V, Kemmerling B, Schulze-Lefert P, Somerville SC, Panstruga R (2006). Conserved requirement for a plant host cell protein in powdery mildew pathogenesis. Nat Genet.

[B25] Kobae Y, Sekino T, Yoshioka H, Nakagawa T, Martinoia E, Maeshima M (2006). Loss of AtPDR8, a Plasma Membrane ABC Transporter of Arabidopsis thaliana, Causes Hypersensitive Cell Death upon Pathogen Infection. Plant Cell Physiol.

[B26] Stein M, Dittgen J, Sanchez-Rodriguez C, Hou BH, Molina A, Schulze-Lefert P, Lipka V, Somerville S (2006). Arabidopsis PEN3/PDR8, an ATP Binding Cassette Transporter, Contributes to Nonhost Resistance to Inappropriate Pathogens That Enter by Direct Penetration. Plant Cell.

[B27] Mentewab A, Stewart CN (2005). Overexpression of an Arabidopsis thaliana ABC transporter confers kanamycin resistance to transgenic plants. Nat Biotechnol.

[B28] Bovet L, Feller U, Martinoia E (2005). Possible involvement of plant ABC transporters in cadmium detoxification: a cDNA sub-microarray approach. Environ Int.

[B29] Lee M, Lee K, Lee J, Noh EW, Lee Y (2005). AtPDR12 Contributes to Lead Resistance in Arabidopsis. Plant Physiol.

[B30] Klein M, Perfus-Barbeoch L, Frelet A, Gaedeke N, Reinhardt D, Mueller-Roeber B, Martinoia E, Forestier C (2003). The plant multidrug resistance ABC transporter AtMRP5 is involved in guard cell hormonal signalling and water use. Plant J.

[B31] Suh SJ, Wang YF, Frelet A, Leonhardt N, Klein M, Forestier C, Mueller-Roeber B, Cho M, Martinoia E, Schroeder J (2007). The ATP binding cassette transporter AtMRP5 modulates anion and Ca^2+ ^channel activities in Arabidopsis guard cells. J Biol Chem.

[B32] Martinoia E, Klein M, Geisler M, Bovet L, Forestier C, Kolukisaoglu U, Muller-Rober B, Schulz B (2002). Multifunctionality of plant ABC transporters – more than just detoxifiers. Planta.

[B33] Lu YP, Li ZS, Rea PA (1997). AtMRP1 gene of Arabidopsis encodes a glutathione S-conjugate pump: Isolation and functional definition of a plant ATP-binding cassette transporter gene. Proc Natl Acad Sci USA.

[B34] Bovet L, Eggman T, Meylan-Bettex M, Polier J, Krammer P, Marin E, Feller U, Martinoia E (2003). Transcript levels of AtMRPs: induction of AtMRP3. Plant Cell Environ.

[B35] Leonhardt N, Vavasseur A, Forestier C (1999). ATP binding cassette modulators control abscisic acid-regulated slow anion channels in guard cells. Plant Cell.

[B36] Gaedeke N, Klein M, Kolukisaoglu U, Forestier C, Muller A, Ansorge M, Becker D, Mamnun Y, Kuchler K, Schulz B, Mueller-Roeber B, Martinoia E (2001). The Arabidopsis thaliana ABC transporter AtMRP5 controls root development and stomata movement. EMBO J.

[B37] Klein M, Geisler M, Suh SJ, Kolukisaoglu HU, Azevedo L, Plaza S, Curtis MD, Richter A, Weder B, Schulz B, Martinoia E (2004). Disruption of AtMRP4, a guard cell plasma membrane ABCC-type ABC transporter, leads to deregulation of stomatal opening and increased drought susceptibility. Plant J.

[B38] Kolukisaoglu HU, Bovet L, Klein M, Eggmann T, Geisler M, Wanke D, Martinoia E, Schulz B (2002). Family business: the multidrug-resistance related protein (MRP) ABC transporter genes in Arabidopsis thaliana. Planta.

[B39] Herbette S, Taconnat L, Hugouvieux V, Piette L, Magniette ML, Cuine S, Auroy P, Richaud P, Forestier C, Bourguignon J, Renou JP, Vavasseur A, Leonhardt N (2006). Genome-wide transcriptome profiling of the early cadmium response of Arabidopsis roots and shoots. Biochimie.

[B40] Kawashima CG, Berkowitz O, Hell R, Noji M, Saito K (2005). Characterization and expression analysis of a serine acetyltransferase gene family involved in a key step of the sulfur assimilation pathway in Arabidopsis. Plant Physiol.

[B41] Harms K, von Ballmoos P, Brunold C, Höfgen R, Hesse H (2000). Expression of a bacterial serine acetyltransferase in transgenic potato plants leads to increased levels of cysteine and glutathione. Plant J.

[B42] Freeman JL, Persans MW, Nieman K, Albrecht C, Peer W, Pickering IJ, Salt DE (2004). Increased glutathione biosynthesis plays a role in nickel tolerance in thlaspi nickel hyperaccumulators. Plant Cell.

[B43] Wirtz M, Hell R (2007). Dominant-negative modification reveals the regulatory function of the multimeric cysteine synthase protein complex in transgenic tobacco. Plant Cell.

[B44] Gayet L, Picault N, Cazale AC, Beyly A, Lucas P, Jacquet H, Suso HP, Vavasseur A, Peltier G, Forestier C (2006). Transport of antimony salts by Arabidopsis thaliana protoplasts over-expressing the human multidrug resistance-associated protein 1 (MRP1/ABCC1). FEBS J.

[B45] Sauge-Merle S, Cuine S, Carrier P, Lecomte-Pradines C, Luu DT, Peltier G (2003). Enhanced toxic metal accumulation in engineered bacterial cells expressing Arabidopsis thaliana phytochelatin synthase. Appl Environ Microbiol.

[B46] Tusnady GE, Simon I (2001). The HMMTOP transmembrane topology prediction server. Bioinformatics.

